# Toxoplasma gondii seroprevalence and risk factors of cats in the Budapest area

**DOI:** 10.1556/1886.2024.00079

**Published:** 2024-11-04

**Authors:** Miklós Pál Dunay, Dorottya Zólyomi, Eszter Gulyás, Ildiko Rita Dunay

**Affiliations:** 1 University of Veterinary Medicine, Budapest, István u. 2, 1078 Budapest, Hungary; 2 Medical Faculty, Institute of Inflammation and Neurodegeneration, Otto von Guericke University, Leipziger Straße 44, D-39120 Magdeburg, Germany; 3 German Center for Mental Health (DZPG), Center for Intervention and Research on Adaptive and Maladaptive Brain Circuits Underlying Mental Health (C-I-R-C), Halle-Jena-Magdeburg, Germany

**Keywords:** Toxoplasma gondii, cat, seroprevalence

## Abstract

This study aimed to survey the current distribution of *Toxoplasma gondii* (*T. gondii*) seropositivity within the cat population in Budapest area. Therefore, blood samples of 123 cats aged 0.5–18 years were collected. The measurements were performed by the commercial ID Screen® Toxoplasmosis Indirect Multi-species ELISA kit. The results indicate an overall 31.7% of seropositivity, which was significantly increasing with age. A correlation was also detected between the outdoor lifestyle and *T. gondii* infection. A significantly higher proportion of cats living outdoors were seropositive (38.8%) compared to those living indoor (18.6%) (*P* = 0.022). Finally, our study indicates a lower *T. gondii* seropositivity rate in cats compared to previous studies from Hungary, as well as from other European regions.

## Introduction

Cats and other Felidae family members are the definitive hosts of the zoonotic apicomplexan parasite *Toxoplasma gondii* [1]. The parasite has a complex life cycle, requiring multiple hosts during its developmental stages. A number of warm-blooded herbivorous and omnivorous animals, including humans, can serve as intermediate hosts. Once bradyzoites are formed in the infected host, intracellular tissue cysts persist lifelong with unique tropism for striated muscle cells and neurons [2]. The sexual developmental stage is restricted to the feline carnivorous host [3]. The exclusive linoleic acid metabolism is proposed to be responsible for the expansion of oocysts in the intestinal epithelium of cats, which is excreted into the environment [4]. The major transmission routes are the oral consumption of raw or undercooked meat containing *T. gondii* tissue cysts or food contaminated with oocysts from cat feces. Cats are also commonly infected by hunting rodents and ingesting their infected tissue [5].

The widespread protozoon *T. gondii* is present on all continents, with a varying seroprevalence between 10 and 85% in humans, which increases with age. The global infection status of cats also varies depending on the geographic region, generally estimated at up to 40% [5]. Three clonal lineages of *T. gondii* exist worldwide (Type I, II, and III), and most infections in Europe occur with the type II strains [6]. Cat ownership is potentially linked to an increased risk of diverse neuropsychological conditions proposed by several studies [7]. Moreover, contact with the cat's litter box during pregnancy should be avoided due to the risk of *T. gondii* infection, which is able to damage the fetus or even cause abortion [8].

Clinical manifestations of toxoplasmosis in cats vary; thus, diagnosis of feline toxoplasmosis is often challenging. Most pathologies are linked to the nervous, alimentary and respiratory systems, although the infection may be subclinical either [5]. From a veterinary point of view, it should be noted that immunosuppression due to feline immunodeficiency virus (FIV) and feline leukemia virus (FeLV) infection can lead to clinical toxoplasmosis. Immunosuppressive drug therapies, especially cyclosporine, can also induce clinical toxoplasmosis [9]. For this reason, screening of patients prior to planned cyclosporine therapy is recommended. Feline infectious peritonitis (FIP) can also lead to clinical toxoplasmosis [1, 10]. The clinical manifestations and their severity are also highly variable [11–14].

Our study aimed to assess the current Toxoplasma seropositivity levels in Budapest area and compare this with previous and international literature.

## Materials and methods

The survey was conducted with a total number of 123 cats, where samples were collected between 11.10.2023 and 20.12.2023 in Budapest, at the Small Animal Clinic of the University of Veterinary Medicine (*N* = 38), and in Törökbálint, at the Vahúr-Vet private veterinary clinic (*N* = 85). All cats were residents of Budapest or the surrounding area. All cats came for diagnostic blood sampling and/or procedures involving the insertion of an intravenous cannulae, thus the blood collection required for the study did not cause them unnecessary pain, stress or any distress.

The serological test used requires a minimum of 10 μL of serum. In all cases, blood was collected from the cephalic vein on the forelimb. The blood sample collected in a native Eppendorf tube was centrifuged at 3,000 rpm for 5 min after clotting, and then 0.2 ml of serum was transferred by pipette into two additional Eppendorf tubes. These were stored in a freezer at −80 °C until 05.01.2024. At this time, the first samples were frozen in dry ice and sent to the partner laboratory in Germany (Otto von Guericke University, Medical Faculty, Institute of Inflammation and Neurodegeneration, Leipziger Straße 44, D-39120 Magdeburg), and the second samples were stored as a backup.

*T. gondii* specific IgG antibodies were detected by the commercial ID Screen® Indirect Multi-species ELISA (Enzyme-Linked Immunosorbent Assay) kit (IDScreen, Grabels, France). The test procedure was performed according to the manufacturer's manual. The optical density (OD) was read at 450 nm using a microplate spectrophotometer TECAN™Sunrise™ and the OD values were collected by the manufacturer Xfluor-4 software.

Each cat owner signed a consent form and filled in a detailed questionnaire about the cat's details and living conditions. From the available information, statistical analyses were performed using R software version 4.0.3 (R Core Team, Vienna, Austria) [15]. Pearson's chi-squared (χ2) test was used to evaluate the correlations between T. gondii seropositivity and other factors (age group, sex, residence, etc.) tested. Chi-squared test was also used to exclude associations due to chance. Correlations were considered significant when the sample size was sufficiently large and *P* < 0.05.

### Ethics statement

The examination was carried out in accordance with the rules of the competent authority (Animal Welfare Committee of the University of Veterinary Medicine Budapest) and in compliance with Hungarian and European regulations.

## Results

A total of 123 cats were investigated, of which 31.7% were *T. gondii* seropositive. Table 1 summarizes all the parameters studied, highlighting the correlations regarding seropositivity.

**Table 1.** Correlations between the parameters studied and *T. gondii* seropositivity

Variables	Categories	Seropositive (%)	Seronegative (%)	Total	*P*-value
Sex	Male	17 (28.8%)	42 (71.2%)	59	0.508
	Female	22 (34.4%)	42 (65.6%)	64	
Neutered	Yes	22 (36.1%)	39 (63.9%)	61	0.303
	No	17 (27.4%)	45 (72.6%)	62	
Breed	European Shorthair	36 (33.6%)	71 (66.4%)	107	0.232
	Other	3 (18.8%)	13 (81.2%)	16	
Age	Young	12 (18.2%)	54 (81.8%)	66	0.00232*
	Middle-aged	17 (45.9%)	20 (54.1%)	37	
	Old	10 (50.0%)	10 (50.0%)	20	
Lifestyle	Outdoors	31 (38.8%)	49 (61.2%)	80	0.0221*
	Indoors	8 (18.6%)	35 (81.4%)	43	
Origin	Controlled	6 (18.8%)	26 (81.2%)	32	0.0671
	Non-controlled	33 (36.3%)	58 (63.7%)	91	
Residence	Capital	4 (13.8%)	25 (86.2%)	29	0.0495*
	Near municipalities	30 (36.1%)	53 (63.9%)	83	
	Distant municipalities	5 (45.5%)	6 (54.5%)	11	
Municipality size	Large city	4 (13.8%)	27 (87.1%)	31	0.01*
	Medium-size city	15 (39.5%)	23 (60.5%)	38	
	Small town	13 (30.2%)	30 (69.8%)	43	
	Village	7 (63.6%)	4 (36.4%)	11	
Population size	Low	20 (37.0%)	34 (63.0%)	54	0.261
	High	19 (27.5%)	50 (72.5%)	69	
FIV** infection	Yes	2 (50.0%)	2 (50.0%)	4	0.594
	No	22 (36.7%)	38 (63.3%)	60	
FeLV*** infection	Yes	3 (75.0%)	1 (25.0%)	4	0.121
	No	22 (36.1%)	39 (63.9%)	61	
Chronic disease	Yes	10 (34.5%)	19 (65.5)	29	0.739
	No	29 (31.2%)	64 (68.8%)	93	
Raw meat feeding	Yes	10 (33.3%)	20 (66.7%)	30	0.826
	No	29 (31.2%)	64 (68.8%)	93	
Cohousing with other cats	Yes	28 (36.8%)	48 (63.2%)	76	0.12
	No	11 (23.4 %)	36 (76.6%)	47	

*The correlation is significant only at a sufficiently large sample size and *P* < 0.05.

**FIV: Feline Immunodeficiency Virus, ***FeLV: Feline Leukemia Virus.

Bold: The association is statistically significant and direct.

### Sex

Among the cats studied, 59 (47.97%) were males, of which 29 were neutered (49.2% of the males), and 64 (52.03%) were females, of which 32 were neutered (50% of the females). Thus, the total number of cats investigated was 61 neutered (49.6%) and 62 unneutered (50.4%). A higher proportion of unneutered cats were *T. gondii* seropositive (36.1%, 22/61) than neutered cats (27.4%, 17/62) (*P* = 0.303). Female cats (34.4%, 22/64) were more often *T. gondii* seropositive than male cats (28.8%, 17/59) (*P* = 0.5078), but these differences were not significant.

### Breed

Most cats involved in the study (107/123, 86.99%) were European Shorthairs. The remaining individuals (16 in total, 13.01%) included 5 Main Coons (4.07%), 2 British Shorthairs (1.63%), 6 other breeds (Himalayan Persian, Carthusian, Ragdoll, Siamese, Sphynx, and Russian Blue, 0.81% each), while three individuals were a mixture of European Shorthair and Persian (2.44%). 33.6% of the European Shorthair cats were *T. gondii* seropositive (36/107), 18.8% of individuals of other breeds were positive (3/16). There was no significant correlation between breed and *T. gondii* seropositivity.

### Age

**Fig. 1.**
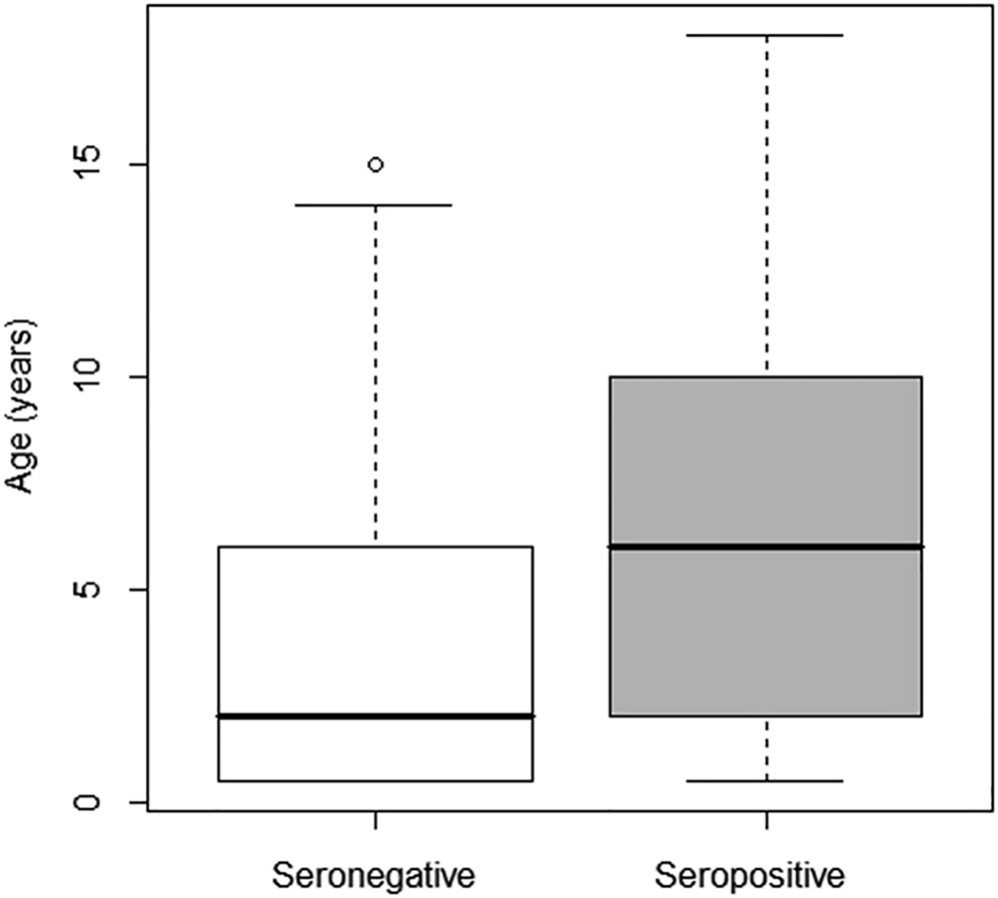
Age distribution of *T. gondii* seronegative and seropositive subpopulations

**Fig. 2.**
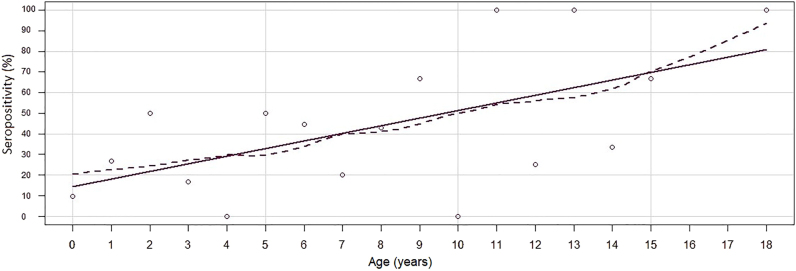
Proportion of *T. gondii* seropositive samples by age

The age of the tested cats ranged from 0.5 to 18 years (mean = 4.77, sd = 4.44, median = 4). Three age groups were formed from the patients: young (less than 5 years), middle-aged (more than 5 years but less than 10 years), and old (more than 10 years). The young individuals were the most represented in the study population (53.66%, 66/123), followed by the middle-aged individuals, which represented 30.08% of the total sample (37/123). The number of old cats was the lowest (16.26%, 20/123). A significant correlation was detected between *T. gondii* seropositivity and age (*P* = 0.002). The prevalence was significantly higher in old (50%, 10/20) and middle-aged cats (54.05%, 20/37) than in young (18.18%, 12/66). The age distribution of the seronegative and seropositive subpopulations is shown in Fig. 1 and the proportion of seropositive samples as a function of age is shown in Fig. 2.

### Lifestyle

As exclusively indoor cats were labelled 43 (34.96%), while the rest were partially or entirely outdoor cats (65.04%, 80/123). A significantly higher proportion of European Shorthair cats were outdoor cats (70.1%, 75/107) than cats of other breeds (31.1%, 5/16) (*P* = 0.002). A significant correlation was detected between *T. gondii* seropositivity and lifestyle as well as housing. A significantly higher proportion of cats living outdoor were positive (38.8%, 31/80) than those living indoor (18.6%, 8/43) (*P* = 0.02205).

### Origin

The highest proportion was of cats were adopted from the street (37.4%, 46/123), followed by cats adopted from a shelter or still living in a shelter (23.6%, 29/123), 18 cats came from a friend (14.6%, 18/123), 9 cats came from a breeder (7.3%, 7/123), 5 cats were born at the owner's home (4.1%, 5/123) and 16 cats were of unknown origin (13%, 16/123). Based on the above, two groups of cats were distinguished: controlled (from an acquaintance, born at home and from a breeding facility) and non-controlled (shelter, street, unknown). The resulting proportion for the former is 26% (32/123) and for the latter 74% (91/123). A lower proportion of cats from controlled environments were *T. gondii* seropositive (18.8%, 6/22) than cats from non-controlled environments (36.3%, 33/91), but the correlation was not significant (*P* = 0.07).

### Residence

In total, the patients came from 20 different municipalities. In the first subdivision, we distinguished between 3 options: Budapest (23.6%, 29/123), municipalities within 100 km of Budapest (67.5%, 83/123) and more distant municipalities (8.9%, 11/123). In the second subdivision, we created four categories: large city (population over 100,000), medium-size city (20–100,000), small town (5–20 000) and village (under 5,000). Similar proportions of patients came from large cities (25.2%, 31/123), medium-size cities (30.9%, 38/123) and small towns (35%, 43/123), but significantly fewer from villages (8.9%, 11/123). Individuals from municipalities far from Budapest had the highest proportion of *T. gondii* seropositive (45.5%, 5/11), followed by those living closer to Budapest (36.1%, 30/83) and then the Budapest population (13.8%, 4/29). Based on *P* = 0.049, the association would be significant, but cannot be accepted due to the low number of individuals from settlements far from Budapest. The highest seropositivity of *T. gondii* was detected in cats from villages (63.6%, 7/11), followed by 30.2% (13/43) in small town cats, 39.5% (15/38) in cats from medium-sized cities and 12.9% (4/31) in cats from large cities. The correlation would be significant (*P* = 0.01), but cannot be accepted due to the low number of individuals from villages.

### Viral coinfections and other pathologies

Among the cats tested for Feline Leukemia Virus (FeLV) (52.8%, 65/123), 4 were positive (6.2%, 4/65), 61 cats were known negative (93.9%, 61/65). In the remaining cases, the infection status was unknown (47.2%, 58/123). Among the individuals in the population with known Feline Immunodeficiency Virus (FIV) status (52%, 64/123), 4 cats were positive (6.3%, 4/64) and 60 cats were negative (93.8%, 60/64). In the remaining cases, the infection status was unknown (48%, 59/123). Among the cats, several individuals (23.8%, 29/123) were suffering from some chronic disease. These included liver and kidney failure, lymphoma, allergies, FIP (Feline Infectious Peritonitis), FCV (Feline Calici Virus) infection, Feline Herpes Virus (FHV) infection, Feline Idiopathic Cystitis (FIC), Feline Odontoclastic Resorptive Lesion (FORL). One FIV positive and one FeLV positive cat were included in this group with other chronic diseases. The other 91 cats (74%, 91/123) did not have any known chronic diseases. No significant correlation was found between FIV infection and *T. gondii* seropositivity (*P* = 0.594), although a higher proportion of FIV positive cats were Toxoplasma infected (50% 2/2) than negative cats (36.7%, 22/60). The sample size is too small to be significant. The same could be said for FeLV infection (*P* = 0.121). 75% of FeLV positive cats (3/4) were *T. gondii* seropositive, while 36.1% of the negative group tested positive (22/61), but the number of cases is too low to establish a significant association. Cats with other chronic diseases did not have significantly higher rates of *T. gondii* infection. A similar proportion of chronically ill cats (34.5%, 10/29) and healthy cats (31.1%, 29/93) were *T. gondii* seropositive.

### Nutrition

The questionnaire included a particular inquiry about feeding raw meat. The frequency of feeding raw meat was not covered. Based on the results received, 24.4% (30/123) of the cats had been fed raw meat, while 75.6% (to the knowledge of the owner) had not yet eaten raw meat (93/123). There was no significant association between raw meat consumption and *T. gondii* seropositivity. 33.3% of the cats that also consumed raw meat became Toxoplasma positive (10/30), 31.2% of the cats that did not consume raw meat became seropositive (29/93) (*P* = 0.826).

### Cohousing with other pets

In total, 61.8% of cats lived with other cats (76/123), 38.2% were single cats (47/123). Cats kept alone were seropositive at a lower rate (23.4% 11/47) than cats kept with other cats (36.8%, 28/76), but no significant correlation was detected (*P* = 0.12).

## Discussion

This study aimed to determine the current seropositivity rate of *T. gondii* in the cat population living in the Budapest area. Seroprevalence in cats reflects the parasite's presence in the environment and, eventually, the risk of human infections. The results indicate a 31.7% *T. gondii* seropositivity investigating 123 individual cats. Previous surveys published in the last 10 years about *T. gondii* infection in cats in Greece [16], Estonia [17], Cyprus [18], Poland [19, 20], Switzerland [21], Turkey [22], Spain [23, 24], Italy [25], Portugal [26], Norway [27] and Finland [28] have reported a seroprevalence ranging from 20.8% to 68.8%. In Cyprus the prevalence in cats was 32.3% measured by ELISA [18]. In Poland, the percentage of *T. gondii* seropositive cats was 49.74% [20]. Previous studies on the South American continent indicate higher *T. gondii* seroprevalence values in humans due to mainly waterborne infection. However, a recent study from Panama reported only a 21.93% *T. gondii* seropositivity in cats [29] and in Brazil up to 15.2 % [30]. In Greece, 20.8% positivity was detected recently [16]. A systematic review published in 2020 reported an overall seroprevalence of *T. gondii* in cats of 67% (95% CI 58–75%) in Europe [31].

A previous report conducted in Hungary in 2007 investigated the prevalence of *T. gondii* and Neospora caninum infection in cats using IFAT (ImmunoFluorescence Antibody Test). The results indicated a prevalence of 47.6% (157/330) of *T. gondii* seropositivity in the cat population studied. A significant correlation (*P* = 0.01) was identified between sex and *T. gondii* seropositivity, with a higher prevalence of infection observed in female cats (53.3%, 104/195) compared to males (39.3%, 53/135). Additionally, an association was noted between the urban, suburban, or rural lifestyle of cats and *T. gondii* seropositivity. The study found that the prevalence of infection was significantly higher (*P* = 0.003) in rural cats (61.3%, 103/168) than in urban or suburban cats (39.1%, 54/162). Moreover, the proportion of positive individuals was found to increase with age, in line with the results of our study [1].

In the current study, the seroprevalence of *T. gondii* was found to be significantly higher in older (50%, 10/20) and middle-aged cats (54.1%, 20/37) when compared to young ones (18.2%, 12/66). A significant correlation was detected between *T. gondii* seropositivity and age (*P* = 0.002). This finding is in agreement with the previously published results [10, 11, 13–15, 17, 20–22] and can be explained by the longer exposure time to the parasite in older individuals. In line with previous studies [13, 15], we also found a correlation between outdoor lifestyle and *T. gondii* infection. A significantly higher proportion of outdoor cats were positive (38.8%, 31/80) than indoor cats (18.6%, 8/43) (*P* = 0.00205). Certain variables, such as residence and municipality size, had an impact on the result not directly but indirectly through outdoor lifestyle.

Further significant correlations are expected to emerge from the extension of the study by increasing the sample size. Additionally, the distribution of clonal lineages in Hungary has not yet been investigated and should be assessed in the future. In summary, our screening of the current *T. gondii* seropositivity rates in cats in Hungary indicates a decreased prevalence of the infection than previously detected.
